# POLR3-related leukodystrophy: How do mutations affecting RNA polymerase III subunits cause hypomyelination?

**DOI:** 10.12703/r/10-12

**Published:** 2021-02-05

**Authors:** Benoit Coulombe, Alexa Derksen, Roberta La Piana, Bernard Brais, Marie-Soleil Gauthier, Geneviève Bernard

**Affiliations:** 1 Department of Translational Proteomics, Institut de Recherches Cliniques de Montréal, Montréal, QC, Canada; 2 Department of Biochemistry and Molecular Medicine, Université de Montréal, Montréal, QC, Canada; 3 Child Health and Human Development Program, Research Institute of the McGill University Health Centre, Montréal, QC, Canada; 4 Department of Neurology and Neurosurgery, McGill University, Montréal, QC, Canada; 5 Department of Neurology and Neurosurgery, Montreal Neurological Institute, McGill University, Montréal, QC, Canada; 6 Department of Diagnostic Radiology, McGill University, Montréal, QC, Canada; 7 Department of Human Genetics, McGill University, Montréal, QC, Canada; 8 Department of Pediatrics, McGill University, Montréal, QC, Canada; 9 Department of Specialized Medicine, Division of Medical Genetics, McGill University Health Center, Montréal, QC, Canada

**Keywords:** leukodystrophy, RNA polymerase III, genetic disease, protein complex assembly, myelination

## Abstract

Hypomyelinating leukodystrophies are a group of genetic disorders characterized by insufficient myelin deposition during development. A subset of hypomyelinating leukodystrophies, named RNA polymerase III (Pol III or POLR3)-related leukodystrophy or 4H (Hypomyelination, Hypodontia and Hypogonadotropic Hypogonadism) leukodystrophy, was found to be caused by biallelic variants in genes encoding subunits of the enzyme Pol III, including POLR3A, POLR3B, POLR3K, and POLR1C. Pol III is one of the three nuclear RNA polymerases that synthesizes small non-coding RNAs, such as tRNAs, 5S RNA, and others, that are involved in the regulation of essential cellular processes, including transcription, translation and RNA maturation. Affinity purification coupled with mass spectrometry (AP-MS) revealed that a number of mutations causing POLR3-related leukodystrophy impair normal assembly or biogenesis of Pol III, often causing a retention of the unassembled subunits in the cytoplasm. Even though these proteomic studies have helped to understand the molecular defects associated with leukodystrophy, how these mutations cause hypomyelination has yet to be defined. In this review we propose two main hypotheses to explain how mutations affecting Pol III subunits can cause hypomyelination.

Leukodystrophies are a heterogeneous group of genetically determined disorders characterized by abnormal white matter on brain imaging^1,2^. The white matter’s major component is represented by myelin that contains mainly lipids and proteins in a ratio of 2.5 to 3:1. Given myelin’s central role in white matter composition, leukodystrophies are classified as hypomyelinating and non-hypomyelinating on the basis of magnetic resonance imaging characteristics^2^, depending on whether the principal problem appears to be a lack of myelin deposition during development or altered myelin homeostasis. Historically, hypomyelinating leukodystrophies have been considered disorders caused by mutations in genes encoding myelin protein constituents such as proteolipid protein and myelin basic protein. In the last decade, it has become clear that mutations in genes encoding proteins important for transcription and translation also lead to hypomyelinating leukodystrophies. A subset of leukodystrophies named RNA polymerase III (Pol III)-related leukodystrophy or 4H (hypomyelination, hypodontia, and hypogonadotropic hypogonadism) leukodystrophy (MIM 607694, 614381)^3^ was found to be caused by biallelic pathogenic variants in genes encoding specific subunits of the enzyme Pol III, namely POLR3A, POLR3B, POLR3K, and POLR1C^4–9^. Figure 1 presents a structural model of Pol III, where these four (out of 17) subunits are highlighted, as is its DNA-binding active site. We suspect that mutations in genes encoding other Pol III subunits and other proteins important for transcription and translation are also leukodystrophy-causative, and next-generation sequencing, combined with functional experiments, is likely to identify them.

**Figure 1.**
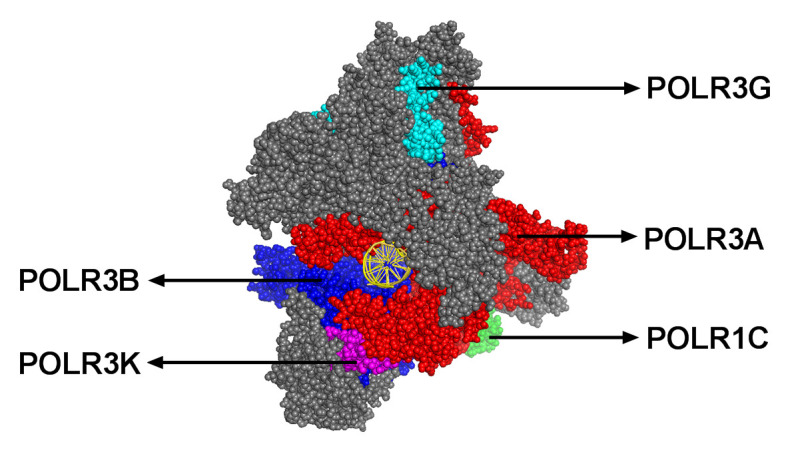
Side view of the cryogenic electron microscopy structure of the *Saccharomyces cerevisiae* RNA polymerase III open DNA complex (Protein Data Bank code: 6EU1)^25^. Template and non-template strands of DNA are shown in yellow (perpendicular to the screen). Human leukodystrophy-causative subunits POLR3A, POLR3B, POLR1C, and POLR3K are labelled. POLR3G, which is homologous to POLR3GL, is also indicated. All other subunits are in gray.

What was initially described as 4H syndrome^10^ was renamed POLR3-related leukodystrophy when the first two genes (*POLR3A* and *POLR3B*) were identified^3–5^. As the disease spectrum widens^11–17^, with hypomyelination not always present, and mutations in *POLR3* genes being found to explain other previously described disorders, they are collectively increasingly referred to as POLR3-related disorders^11–13,18–22^. With this new definition, one other POLR3 gene, *POLR3GL*, was added to the list of genes with biallelic mutations causing disorders with overlapping manifestations but without a leukodystrophy^23,24^*.* POLR3GL is an interesting Pol III subunit as it is homologous to POLR3G. They independently associate with the enzyme; POLR3G is found in undifferentiated cells and is shown to play a role in maintaining the pluripotent state, and POLR3GL is expressed ubiquitously^26–29^. As a consequence, two forms of Pol III can be purified and the specific role of each form in disease remains to be studied.

Pol III is one of the three nuclear RNA polymerases, the two others being Pol I and Pol II, and it synthesizes small non-coding RNAs—such as tRNAs, 5S RNA, 7SK RNA, and U6 RNA—that are involved in the regulation of essential cellular processes, including transcription, RNA processing, and translation^30^. Pol I synthesizes large rRNA while Pol II synthesizes all mRNA. Each polymerase possesses its own set of accessory factors that are required to transcribe their specific set of target genes. One would expect that affecting the activity of Pol III would have a general effect on gene expression and cell function rather than a specific effect on myelin formation or dental and pituitary development observed in patients. The pathophysiological link between mutations in genes encoding Pol III subunits and the involvement of such specific tissues remains unknown.

Affinity purification experiments coupled with mass spectrometry performed in HEK293 cells as a model system, in which wild-type and mutated subunits were compared, revealed that a number of mutations causing POLR3-related leukodystrophy impair proper assembly/biogenesis of Pol III, often causing a retention of the unassembled subunits in the cytoplasm as revealed by immunofluorescence and biochemical fractionation of cell lysates^8,31^. Even though these studies have helped elucidate the molecular defects associated with leukodystrophy, how these mutations cause hypomyelination has yet to be determined.

In our opinion, two main hypotheses can be favored at this stage to explain how mutations affecting RNA Pol III subunits can cause hypomyelination.****

## 1. Specific key POLR3 target(s) essential for myelin biogenesis

Some authors have hypothesized that defects in Pol III function likely affect a key yet-unidentified component of the myelin biogenesis or maintenance apparatus. For instance, Choquet *et al*. found that the M852V mutation in POLR3A specifically downregulates expression of *BC200*, a gene encoding a brain cytoplasmic regulatory RNA^32^. So far, a function for BC200 in regulating myelination has not been established conclusively.

## 2. Globally reduced transcription and translation at a crucial neurodevelopmental milestone

Another hypothesis is based on the evidence that the brain is particularly vulnerable during development. More specifically, oligodendrocytes (OLs) and their progenitors need a large amount of protein to be produced in order to mature, expand their processes, and myelinate the brain. Selective vulnerability of late OLs progenitors is a well-documented phenomenon in pediatric neuropathology^33^. Defects in Pol III transcription, by impairing the production of components of the translation machinery (tRNA and 5S rRNA), may have a deleterious effect on this particular process. This hypothesis is supported by the fact that other disorders where the pathophysiological mechanisms involve abnormal translation are also associated with hypomyelination^34,35^. Interestingly, mutations targeting genes for aminoacyl-tRNA synthetases (ARSs) (see, for example, Mendes *et al*.^34^ and references therein) are also causative of a hypomyelinating leukodystrophy. ARSs play a central role in translation by charging the various tRNAs with their cognate amino acids.

It is also possible that a not-yet-understood biochemical pathway is involved in POLR3 function. Future studies should help to assess this idea.

Altogether, studies so far have characterized Pol III defects produced by mutations that are causative of various overlapping rare diseases. However, the mechanisms linking these to hypomyelination and other phenotypic characteristics (for example, hypodontia and hypogonadotropic hypogonadism) are still unknown at this stage. Clearly, additional studies are required to better elucidate the pathophysiology of POLR3-related disorders.
